# Effects of backpack load and position on body strains in male schoolchildren while walking

**DOI:** 10.1371/journal.pone.0193648

**Published:** 2018-03-21

**Authors:** Yi-Lang Chen, Ying-Cen Mu

**Affiliations:** 1 Department of Industrial Engineering and Management, Ming Chi University of Technology, New Taipei, Taiwan; 2 Department of Industrial Design, Chang Gung University, Touyuan, Taiwan; University of L’Aquila, ITALY

## Abstract

Data regarding the effects of backpack carriage on children’s body strains while walking are limited. This study measured the body posture, muscle activation, and subjective discomfort scores of 12 male schoolchildren (age: 12.3 (range 12.1–13.0) y, height: 151.3 (range 144.2–154.6) cm, weight: 46.6 (range 43.6–49.7) kg) carrying backpacks weighing 5%, 10%, and 15% of their respective body weights (BWs) and walking for 10 min on a treadmill. For each load, three positions along the spinal column (T7, T12, and L3) were examined. Participants carrying a backpack weighing 15% of BW exhibited higher head flexion, trunk flexion, and corresponding muscle activation, and a lower lumbosacral angle compared with those carrying loads of 5% and 10% of BW. The waist received the highest discomfort scores when the backpacks were carried at the L3 position. Conversely, the discomfort rating for the neck and shoulders where the highest when the backpack was at the T7 position; this high backpack position also caused more head flexion than the other two positions. For the musculoskeletal health of children, the findings suggest that carrying a school backpack weighing 15% of BW should be avoided, and carrying at the T12 position may be recommended for schoolboys.

## Introduction

Most schoolchildren in developed countries carry backpacks. Previous studies have demonstrated that daily physical stresses associated with carrying backpacks cause significant forward lean of the head and trunk [1–3] and changes in spinal curvature [4,5]. Neuschwander et al. [6] found that an increase in backpack load significantly compresses lumbar disc heights measured in the midline sagittal plane. Daily intermittent postural adaptations are assumed to result in pain and disabilities in schoolchildren [7–9].

One of the crucial factors of backpack carriage is the weight. In Australia, the average load carried by children was found to be 5.3 kg, or approximately 10% of their mean body weight (BW) [10]. In a study in the United States, the mean weight of school bags was 17% of the students’ mean BW [11]. Dockrell et al. [12] surveyed 529 children in Ireland and found that the mean schoolbag weight was 12.6% of BW, and only 29.9% of children carried schoolbags that were less than or equal to 10% of BW. Negrini et al. [13] found that the average load carried by Italian schoolchildren was 22.0% of BW. In summary, children load their backpacks at between 10% and 22% of BW [14]; however, the recommended backpack weight limit for schoolchildren varies from 5% to 20% of BW [15]. The literature remains inconclusive.

In addition to backpack weight, attention has been given to the height of the backpack’s center of gravity (CG). The position of the backpack is crucial for determining the proper load-carriage method and for the ergonomic design of schoolbags. In general, a high position of the load is recommended [4]. Devroey et al. [16] found that carrying a backpack positioned at the lumbar region led to higher spinal flexion than carrying a backpack positioned at the thoracic region; however, their participants showed a preference for the lumbar placement. Grimmer et al. [17] investigated the sagittal postural changes when a backpack is carried with its CG at T7, T12, and L3 positions, and suggested that backpacks should be positioned at the waist or hip level. Suggestions regarding the optimal carrying position are contradictory.

Although backpack carriage is a dynamic activity, studies have typically performed backpack-carrying tests on subjects in static standing postures. Some studies have collected data from participants walking for short durations while carrying backpacks, but these studies either included college-aged adults [17] or did not evaluate backpack placement [18–20]. Some studies have recruited schoolchildren to walk under various combinations of weights and positions, but they have focused on specific issues, such as shoulder contact pressure [21]. This study comprehensively evaluated backpack carriage using posture analyses, muscle activation, and subjective discomfort scores. We hypothesized that a more acceptable combination of the weight and the position of backpacks for schoolboys may exist when considering the body strains.

## Materials and methods

### Participants

This study recruited 12 male schoolchildren and paid them at an hourly rate. Recruitment information was announced on the bulletin board of Ming Chi Elementary School (New Taipei, Taiwan) during January 1–20, 2013. The volunteers were then interviewed and informed of the details of the test procedure. The mean (range) age, height, and BW of the study participants were 12.3 (12.1–13.0) y, 151.3 (142.2–154.6) cm, and 46.6 (43.6–49.7) kg, respectively. All participants were right-hand dominant and were healthy with no reported musculoskeletal problems or back pain in the 12-month period prior to the study. The experimental procedures were approved by the Ethics Committee of Chang Gung Memorial Hospital (Taiwan, No. 98-3653A3), and informed and written consent was obtained from all the study participants and their parents prior to beginning the experiment.

### Posture analysis

**Fig 1:**
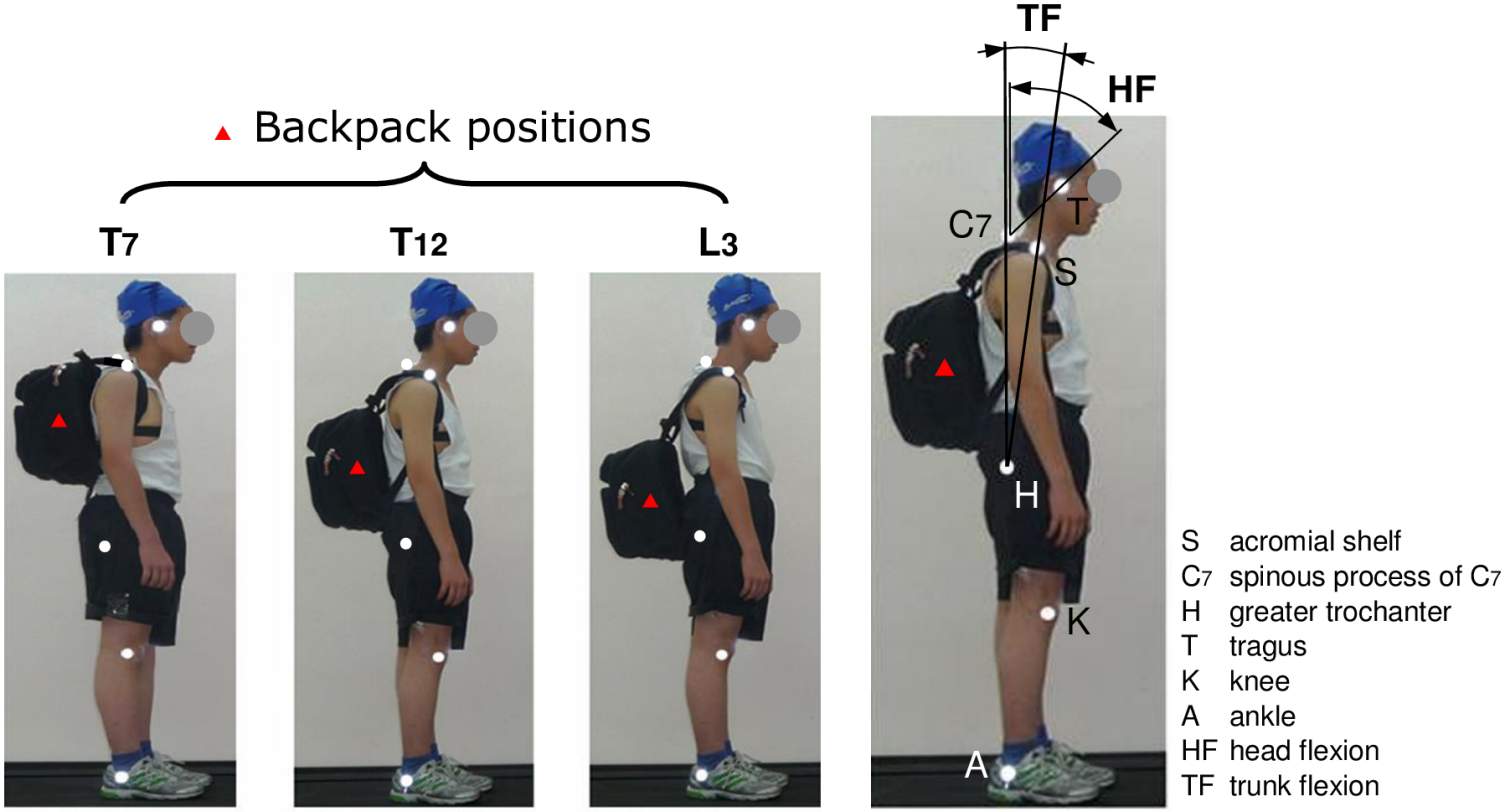
Backpack positions and body postures examined.

The backpack-carrying postures of the participants were recorded while they walked on a treadmill (CS-5728, Chanson, Taipei) for 10 min with various combinations of loads and positions. Because body joint angles in the sagittal plane were analyzed in this study, two-dimensional kinematics data were collected. Before data collection, six adhesive reflective markers were attached to each participant’s anatomical landmarks (i.e., the tragus, acromial shelf, spinous process C7, femoral greater trochanter, knee, and ankle) on the dominant side (Fig 1). The anatomical landmarks of each participant were identified by an orthopedic surgeon with 15 years of experience. All markers, except the hip marker, were attached to the skin. The hip marker was attached to participants’ shorts and fixed by a tightening strap to prevent deviation. The experimenter also ensured that all markers remained at the exact landmark positions during the test. The angles of head flexion (i.e., craniocervical angle) and trunk flexion (formed by the vertical line linking the acromial shelf and the hip) were measured. Because the interference from the backpack hindered measurement during the test, each participant’s lumbosacral angle (defined as the angle between the superior surface of the first lumbar vertebrae (L1) and first sacral vertebrae (S1)) was estimated using the following equations developed by Chen [22] for predicting the vertebral inclination of the lumbar spine:

$$L_{1}\;=\;1.04\times trunk-11.16\;(R^{2}\;=\;0.969)$$

$$S_{1}\;=\;34.65\times 1.26^{\left(trunk/30\right)}\;(R^{2}\;=\;0.916)$$

where “trunk” is the flexion angle from the upright position (in degrees). The lumbosacral angle can then be obtained by determining the angle formed by L1 and S1, as defined by Chen [23].

### Electromyography

In this study, a wireless electromyography (EMG) device (TeleMyo 2400, Noraxon, Scottsdale, USA) was used to measure activation in three muscle groups (trapezius, latissimus dorsi, and erector spinae). Because the kinematics data were analyzed as the body posture was photographed in the sagittal plane, muscle group activation was measured on the left side of each participant. The EMG electrodes were attached on the opposite side of the joint markers because some participants felt relatively uneasy and unbalanced when all attachments were attached on the same side during walking. We placed pairs of Ag/AgCl surface electrodes (lead-off area, 10 × 10 mm^2^; center-to-center electrode distance, 45 mm) parallel to the muscles following a standard preparation procedure; prior to electrode attachment on the skin, the area was shaved and cleaned with alcohol. Bipolar surface electrodes were attached to each participant’s skin with adhesive tape to avoid artifacts. The following electrode locations were used: trapezius, the midpoint between C7 and the acromion [24]; latissimus dorsi, lateral to T9 over the muscle belly [25]; erector spinae, 3 cm lateral to the L3 spinous process [25].

Prior to EMG recording, participants completed the standardized muscle-specific maximal voluntary contractions (MVCs) to normalize signals measured during backpack trials [26]. Each muscle MVC test was held for at least 5 s for further analysis. The MVC testing procedure was performed as described by McGill [25], and the MVC techniques for three muscle groups were adapted from Troiano et al. [24] and Vera-Garcia et al. [27]. EMG data were collected for the final 10 s of the 10-min walking period. The electrical signals collected from both MVC tests and backpack trials were filtered with high and low frequencies of the analog band pass filter (20–600 Hz) used before the signals were sampled (1200 Hz). The sampled signals were then fully rectified and processed to produce integrated EMG (IEMG) data [28,29]. In this study, a normalization procedure was performed to compare the IEMG data from the experimental trial with the twofold MVC IEMG data in an identical interval of 10 s. All muscle activation values were presented as percentages of the IEMG data of the MVC.

### Subjective discomfort scores

In this study, subjective assessments were performed using a continuous visual analog scale. The scale was 100 mm in length and was modeled after the comfort scales developed by Mundermann et al. [30]. The visual analog scale is a reliable means of perception assessment and is more precise than ordinal scales [30]. The left end of the scale was labeled “no discomfort at all” and the right end was labeled “extreme discomfort.” The participants used a pen to mark locations along the scale that most accurately represented their discomfort after a trial. The distance was measured from the “no discomfort at all” anchor to the location of a mark, and these distance data were used for analysis. The levels of discomfort experienced by each participant in the neck and shoulders, back, and waist were rated. After each participant had walked with the backpack for 10 min, the participant was immediately asked to provide discomfort scores for the body sites. The 10-min walking duration approximates the common travel time between home and school for Taiwanese children.

### Experimental design and procedures

During the experiment, the participants wore clothing suitable for sports (Fig 1). Prior to the experiment, the participants were required to warm up for 5 min to become familiar with walking on the treadmill. During the test, the velocity of the treadmill was set at 1.3 m/s, which can be considered a normal walking pace [31]. The testing backpack was a commercial double-strap backpack (dimensions 45 × 30 × 20 cm^3^, net weight 0.8 kg, No.1998-2002, UnMe, A-Star CO., LTD, Taiwan). Its CG was controlled using a custom-made wooden frame placed inside the backpack. The weights (equivalent to 5%, 10%, and 15% of each participant’s BW) were set using small lead blocks (dimensions 5 × 5 × 1.2 cm^3^). The weights for testing were obtained from previous studies [5,16,32]. The load of 20% of BW was not considered as a testing level because it was too heavy for some participants. Moreover, carrying 20% of BW is significantly correlated with shoulder and back pain in children [14]. The lengths of the shoulder straps for each position were adjusted by the participants to an assigned position. Thereafter, the lengths were fixed for each position throughout the experiment. The three backpack positions examined in this study were T7, T12, and L3 [16], which were selected on the basis of the midline of the vertical length of the backpack (Fig 1). To ensure that the backpack was in the desired position, the experimenter identified the T7, T12, and L3 of each participant to fit the center of backpack at an identical height.

Body posture and EMG data were synchronously collected during the last 10 s of the 10-min walking duration for the nine test combinations. We used a trigger signal to start collection of both motion and EMG data to ensure synchronization. Moreover, a test was also performed without a backpack by each participant for comparison. The average values for body posture and EMG data for the last 10 s were used for analysis. To prevent muscle fatigue, a minimum rest period of 20 min was allowed between successive trials, and each participant was tested for less than 2 h during each half day. We obtained 108 backpack data samples (12 participants × 3 backpack weights × 3 backpack positions) and an additional 12 samples of participants for the no-backpack condition. For each participant, backpack weight and position combinations were applied in a random order. During the test, a camera (Qualisys, MacReflex motion analysis system, Sweden) positioned approximately 5 m from the participant and perpendicular to the sagittal plane, recorded the kinematic marker positions (resolution = 1:30,000 in the camera field of view at 120 Hz and low-pass filtered at 6 Hz).

### Statistical analysis

A two-way repeated-measures analysis of variance (ANOVA) was used to investigate the effects of three backpack weights and three backpack positions, and the Duncan multiple range test (MRT) was used for post hoc comparisons. The Duncan MRT is a widely used procedure for comparing all pairs of means and is very effective at detecting differences between means [33]. In the analyses, a randomized 3 × 3 design was used, and the no-backpack condition was excluded because the position levels were absent in that condition. The effects of the backpack variables on physical indices (posture and muscle activation) were also evaluated with reference to the no-backpack condition. All statistical analyses were conducted using statistical software (SPSS 19.0), and the level of significance was set at 0.05.

## Results

### Body postures

**Fig 2:**
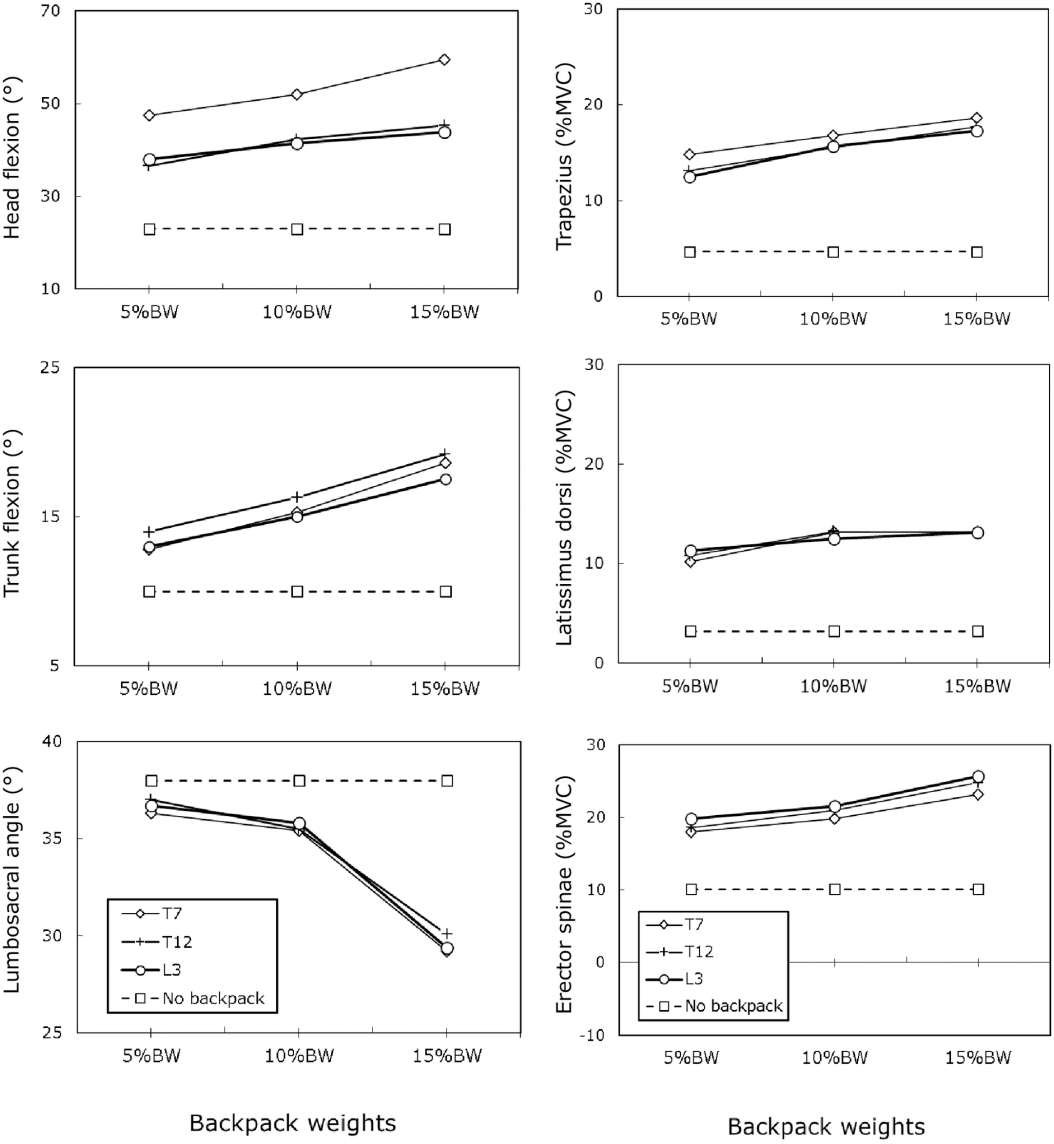
Comparison of body postures and muscle activation associated with backpack weights and positions.

Data of body posture, muscle activation, and discomfort score of all participants are provided for each backpack-carrying combination as supporting information (S1 Dataset). Table 1 shows that head flexion was significantly affected by backpack weight and position (*p* < 0.001). Trunk flexion and lumbosacral angle were also affected by backpack weight, but not by backpack position (*p* < 0.001). All two-factor interactions were not significant. Tables 2 and 3 show the Duncan MRT results of body postures under different backpack weights and positions, respectively. As shown in Table 2, when a participant carried a backpack weighing 5% of BW, head flexion was the smallest (40.8°), whereas the maximum head flexion was observed at 15% of BW (49.5°). A similar trend was observed in trunk flexion. Notably, when carrying a backpack weighing 15% of BW, the participant’s lumbar curvature exhibited less lordosis (29.6°) than when the participant carried lighter backpacks (5% of BW: 36.7° and 10% of BW: 35.5°). The high position (T7) resulted in the highest head flexion (Table 3). This greatest head flexion was approximately 12° greater than that when the backpack was at position T12 or L3. However, trunk flexion and lumbosacral angle did not vary depending on the backpack position. Fig 2 depicts the body postures and a comparison with the no-backpack condition. Head flexion increased when the backpack was at the T7 position. Trunk flexion increased with an increase in backpack weight. Position showed a nonsignificant effect on trunk flexion. The heaviest backpack caused a larger decrease in lumbar curvature than the other two weight levels did.

**Table 1:** Summarized ANOVA results of body postures.

Body postures	Variables	df	F	*p*-value
Head flexion	Backpack weight	2	10.50	<0.001
	Backpack position	2	30.33	<0.001
	Weight×position	4	0.04	0.997
Trunk flexion	Backpack weight	2	20.74	<0.001
	Backpack position	2	2.02	0.139
	Weight×position	4	0.93	0.453
Lumbosacral angle	Backpack weight	2	117.38	<0.001
	Backpack position	2	11.52	0.135
	Weight×position	4	5.32	0.442

**Table 2:** Duncan MRT results of body postures under backpack weight conditions.

Body postures	Backpack weights (%BW)	Samples	Mean (SD)(°)	Duncan groups*
Head flexion	5	36	40.8 (8.9)	A
	10	36	45.2 (9.1)	B
	15	36	49.5 (9.8)	C
Trunk flexion	5	36	13.4 (2.8)	A
	10	36	15.5 (3.6)	B
	15	36	18.3 (3.3)	C
Lumbosacral angle	5	36	36.7 (2.3)	A
	10	36	35.5 (2.8)	A
	15	36	(2.6)	B

*Differing letters within backpack weights indicate significant differences between the means.

**Table 3:** Duncan MRT results of body postures under backpack position conditions.

Body postures	Backpack positions	Samples	Mean (SD) (°)	Duncan groups*
Head flexion	T7	36	53.0 (8.1)	A
	T12	36	41.4 (7.5)	B
	L3	36	41.2 (8.0)	B
Trunk flexion	T7	36	15.5 (3.7)	--
	T12	36	16.6 (3.5)	--
	L3	36	15.1 (4.1)	--
Lumbosacral angle	T7	36	33.3 (2.6)	--
	T12	36	34.4 (3.0)	--
	L3	36	34.1 (2.8)	--

*Differing letters within backpack positions indicate significant differences between the means.

### Muscular activation

Table 4 summarizes the two-way ANOVA results for muscle activation. Only the trapezius and erector spinae muscles were significantly affected by the various backpack weights (*p* < 0.05), whereas the other combinations (including two-factor interactions) were not significantly affected. Furthermore, the Duncan MRT results (Table 5) showed differences in muscle activation for backpack weights of 5% and 10% of BW compared with 15% of BW. Muscle activation was not affected by backpack position (Table 6). Fig 2 illustrates the significant increases in muscle activation in the trapezius and erector spinae muscles with an increase in backpack weight.

**Table 4:** Summarized ANOVA results of muscle activation.

Muscles	Variables	df	F	*p*-value
Trapezius	Backpack weight	2	3.94	0.023*
	Backpack position	2	0.36	0.697
	Weight×position	4	0.01	0.999
Latissimus dorsi	Backpack weight	2	2.60	0.080
	Backpack position	2	0.03	0.968
	Weight×position	4	0.37	0.832
Erector spinae	Backpack weight	2	6.54	0.002**
	Backpack position	2	1.21	0.304
	Weight×position	4	0.03	0.998

*p < 0.05;

**p < 0.01

**Table 5:** Duncan MRT results of muscle activation under backpack weight conditions.

Muscles	Backpack weights (%BW)	Samples	Mean (SD) (%MVC)	Duncan groups*
Trapezius	5	36	13.4 (6.2)	A
	10	36	15.8 (6.9)	AB
	15	36	18.1 (6.9)	B
Latissimus dorsi	5	36	10.8 (2.8)	--
	10	36	12.9 (3.6)	--
	15	36	13.1 (3.3)	--
Erector spinae	5	36	18.9 (5.5)	A
	10	36	20.9 (4.3)	A
	15	36	24.3 (4.6)	B

*Differing letters within backpack weights indicate significant differences between the means.

**Table 6:** Muscle activation values under backpack position conditions.

Muscles	Backpack positions	Samples	Mean (SD) (%MVC)
Trapezius	T7	36	16.7 (6.6)
	T12	36	15.5 (7.2)
	L3	36	15.2 (6.7)
Latissimus dorsi	T7	36	12.2 (4.6)
	T12	36	12.4 (4.4)
	L3	36	12.4 (4.9)
Erector spinae	T7	36	20.7 (7.1)
	T12	36	21.5 (5.8)
	L3	36	22.5 (6.1)

### Subjective discomfort scores

Table 7 shows the effects of backpack weight and position on discomfort scores. Discomfort scores for both the neck and shoulders and the waist were affected by both backpack weight (*p* < 0.05) and position (*p* < 0.001), whereas the back score was affected only by weight. No interaction effect of backpack weight and position was observed. The Duncan MRT result showed that the highest discomfort scores for the neck and shoulders, back, and waist were given when a weight of 15% of BW was carried (Table 8). Unlike how the discomfort score increased with an increase in backpack weight, the effect of backpack position in discomfort score varied depending on the body sites (Table 9). Carrying the backpacks at the T7 position affected neck and shoulder discomfort more than any other position. Conversely, the waist received the highest discomfort scores when the backpacks were carried at the L3 position.

**Table 7:** Summarized ANOVA results of subjective discomfort scores.

Body sites	Variables	df	F	*p*-value
Neck/shoulders	Backpack weight	2	18.95	<0.001
	Backpack position	2	12.02	<0.001
	Weight×position	4	0.49	0.806
Back	Backpack weight	2	5.99	0.004**
	Backpack position	2	0.98	0.378
	Weight×position	4	0.64	0.634
Waist	Backpack weight	2	4.31	0.016*
	Backpack position	2	7.40	<0.001
	Weight×position	4	1.12	0.350

*p < 0.05;

**p < 0.01

**Table 8:** Duncan MRT results of discomfort scores under backpack weight conditions.

Body sites	Backpack weights (%BW)	Samples	Mean (SD)	Duncan groups*
Neck/shoulders	5	36	1.8 (0.7)	A
	10	36	2.8 (1.2)	B
	15	36	4.1 (1.5)	C
Back	5	36	1.8 (0.9)	A
	10	36	2.1 (1.1)	A
	15	36	3.0 (1.3)	B
Waist	5	36	1.4 (0.6)	A
	10	36	1.9 (0.7)	AB
	15	36	2.3 (1.0)	B

*Differing letters within backpack weights indicate significant differences between the means.

**Table 9:** Duncan MRT results of discomfort scores under backpack position conditions.

Body sites	Backpack positions	Samples	Mean (SD)	Duncan groups*
Neck/shoulders	T7	36	4.0 (1.5)	A
	T12	36	2.5 (1.2)	B
	L3	36	2.2 (1.0)	B
Back	T7	36	2.3 (0.9)	--
	T12	36	2.2 (1.2)	--
	L3	36	2.4 (1.1)	--
Waist	T7	36	1.4 (0.7)	A
	T12	36	1.6 (0.6)	A
	L3	36	2.7 (1.3)	B

*Differing letters within backpack weights indicate significant differences between the means.

## Discussion

The results of previous backpack studies lack uniformity in examination of posture alteration and muscle activation, particularly with regard to the weight carried and the position of the weight. Reasons for the lack of uniformity may be differences in participant groups (children vs. adults), testing protocols (dynamic or walking vs. static or standing), or assessment indices (biomechanics, physiology, and psychophysics). To understand the most common backpack problems in schoolchildren, this study investigated body posture, muscle activation, and subjective discomfort scores in male schoolchildren who walked for 10-min periods carrying backpacks with various weight and position combinations.

### Effect of backpack weight

One study suggested that backpacks weighing between 10% and 20% of BW may be acceptable for school children [34]; another report conservatively concluded that the acceptable limit is between 10% and 15% of BW [32]. These recommendations are higher than those suggested by the results of this study. Our results suggest that a backpack weighing 15% of BW should be avoided because of the increased head and trunk flexion, considerably decreased lumbosacral angle, and more notable discomfort experienced compared with a backpacking weighing 10% of BW. A 10% of BW limit was suggested by Kistner et al. [20], who investigated postural compensations and subjective complaints. Our backpack weight findings agree with the results from Kistner et al. [20] and Chow et al. [35].

Devroey et al. [17] concluded that loads of 10% of BW or more are unacceptable because these loads induced significant changes in the EMG, kinematics, and subjective scores in their study. In our study, a significant decrease in lumbar lordotic curvature was observed when backpack weight was increased from 10% (35.5°) to 15% of BW (29.6°), whereas no significant difference was observed between backpack weights of 5% (36.7°) and 10% (35.5°) of BW. The decrease in lumbar lordosis for a backpack weighing 15% of BW may be attributed to the fact that the children made increased efforts to counterbalance excessive external loads. The children were able to maintain normal lumbar posture when carrying lighter backpacks, but found it difficult to maintain normal lumbar lordosis (38.7° in this study) with the heaviest backpacks. The relatively awkward lumbar posture when the backpack load was 15% of BW also corresponded to higher erector spinae activation (Table 5). With increased loading, increased activation of postural muscles (e.g., erector spinae) provided spinal stability [36]. The increased flattening of lumbar lordosis and higher erector spinae activation may increase the load on the back and therefore increase the risk of back injury [37,38]. However, the increased erector spinae activation with increasing weight found in this study contradicts the result of Devroey et al. [17]. They observed a decrease in erector spinae activation with increasing weight (and an increase in abdominal muscle activation), and this was attributed to the load being carried passively. Different carriage patterns may be due to the difference in participant recruitment (12 schoolboys vs. 20 college-aged male and female students). This inconsistency merits further investigation.

### Effect of backpack position

Studies on the influence of backpack position have been somewhat inconclusive. Grimmer et al. [16] claimed that typical school backpacks should be positioned with the CG at waist or hip level. Conversely, Devroey et al. [17] found a trend toward increased spinal flexion and reduced pelvic anteversion when backpacks were carried at lower positions (i.e., lumbar placement) compared with when they were carried at higher positions (i.e., thoracic placement). Macias et al. [21] found that shoulder contact pressures from straps were significantly greater in the low-back condition than in the high-back condition. In our study, neck and shoulder discomfort scores increased as the backpack height increased, and positioning backpacks at the T7 position resulted in a higher increase in forward head flexion than any other conditions. Body discomfort scores were strongly associated with backpack positions except back region. When the backpack was at T7 and L3 height levels, the highest discomfort scores were in the neck and shoulders and the waist, respectively. No differences in discomfort scores were observed for the back region among the three positions. We found that a trade-off backpack position was T12 with various weights carried. Carrying a backpack at the T12 position may lead to a less flexed upper body and less discomfort. Chow et al. [35] performed a study on the effect of backpack load on gait; their backpack was set at the T12 level, and the recommended critical load was 10% of BW. However, their participants were 22 adolescent girls with a mean age of 13.4 y.

### Study limitations

One major limitation of this study is the relatively small and single-sex sample (12 schoolboys with a mean age of 12.3 y) recruited in the test. The results may not be applicable to the female population because the prevalence of lower-back pain caused by backpack carriage may be higher in girls than in boys [9], and changes in carriage posture were shown to be different between girls and boys [39]. In addition, posture and muscle activation were examined only in the sagittal plane, and the maximum backpack weight was 15% of BW. These limitations should be considered before the results are widely applied. Larger and more diverse samples (e.g., schoolgirls) warrant consideration in further investigation.

## Conclusion

This study required participants to carry backpacks weighing 5%, 10%, and 15% of their total BW and walk for 10 min on a treadmill. Each carrying weight was also investigated at three backpack positions. The results show that differences in head and trunk flexion and lumbosacral angle between the no-backpack condition and carrying a load weighing 15% of BW were significant in comparison with differences between the other observed loads. Positioning the backpack near the T12 position may avoid extreme discomfort at the body sites investigated. This study suggests that carrying a load weighing no more than 10% of BW at the T12 position may be acceptable for schoolboys.

## Supporting information

S1 Dataset The body postures, muscle activations, and discomfort scores for each backpack carrying combination. (XLS)
